# The recombined cccDNA produced using minicircle technology mimicked HBV genome in structure and function closely

**DOI:** 10.1038/srep25552

**Published:** 2016-05-13

**Authors:** Xiaoyan Guo, Ping Chen, Xiaohu Hou, Wenjuan Xu, Dan Wang, Tian-yan Wang, Liping Zhang, Gang Zheng, Zhi-liang Gao, Cheng-Yi He, Boping Zhou, Zhi-Ying Chen

**Affiliations:** 1 The Laboratory for Gene and Cell Engineering, Shenzhen Institutes of Advanced Technology, Chinese Academy of Sciences, Shenzhen, 518055, China; 2 Department of Infectious Diseases, The Third Affiliated Hospital of Sun Yat-Sen University, 600 Tian He Road, Guangzhou, 510630, China; 3 Institute of Hepatology, Shenzhen Third People’s Hospital, Shenzhen, 518112, China

## Abstract

HBV covalently closed circular DNA (cccDNA) is drug-resistant and responsible for viral persistence. To facilitate the development of anti-cccDNA drugs, we developed a minicircle DNA vector (MC)-based technology to produce large quantity of recombined cccDNA (rcccDNA) resembling closely to its wild-type counterpart both in structure and function. The rcccDNA differed to the wild-type cccDNA (wtcccDNA) only in that it carried an extra 36-bp DNA recombinant product *attR* upstream of the preC/C gene. Using a procedure similar to standard plasmid production, milligrams of rcccDNA can be generated in common laboratories conveniently. The rcccDNA demonstrated many essential biological features of wtcccDNA, including: (1) undergoing nucleation upon nucleus entry; (2) serving as template for production of all HBV RNAs and proteins; (3) deriving virions capable of infecting tree shrew, and subsequently producing viral mRNAs, proteins, rcccDNA and infectious virions. As an example to develop anti-cccDNA drugs, we used the Crispr/Cas9 system to provide clear-cut evidence that rcccDNA was cleaved by this DNA editing tool *in vitro.* In summary, we have developed a convenient technology to produce large quantity of rcccDNA as a surrogate of wtcccDNA for investigating HBV biology and developing treatment to eradicate this most wide-spreading virus.

Hepatitis B virus (HBV) infection remains a severe burden of public health system. More than 240 million people are chronically infected worldwide with a high risk of progressing to life-threatening liver cirrhosis and hepatocellular carcinoma (HCC)^1^. HBV is the known smallest DNA virus containing a 3.2-kb partially double-stranded relaxed circular DNA (rcDNA) genome^2^. Upon infection, the virus enters the host hepatocytes and releases its rcDNA genome into the nucleus, where the rcDNA is converted to a covalently closed circular DNA (cccDNA)^2^. The cccDNA exists as a stable mini-chromosome in the nucleus and serves as the template for generating all the viral RNA transcripts^2^,^3^. Among them, the pgRNA is encapsidated into the capsid and converted into rcDNA subsequently via a unique protein-primed reverse transcription process^2^,^4^. The majority of the newly formed capsids are enveloped with cellular surface proteins and lipids before being secreted into circulation as virions; but some are dissembled and they releas their rcDNA into the nucleus via a mechanism known as intracellular recycling, this portion of rcDNA was converted into cccDNA to replenish the nuclear cccDNA pool^4^,^5^,^6^,^7^. Due to an ill-defined mechanism, the cccDNA maintains a steady-state population of a few copies per infected hepatocyte^3^,^8^,^9^. It has been documented that the cccDNA, once formed, is able to persist in human liver for years^10^.

As its stability and drug-resistance features, cccDNA is responsible for the persistence of HBV infection^11^. Currently, there is no antiviral drug to cure chronic HBV infection. Although the nucleotide-analogue antiviral drugs are able to efficiently inhibit HBV replication and profoundly shrink the liver cccDNA pool, they fail to completely eliminate the cccDNA. A viral rebound almost inevitably occurs soon after the cessation of treatment, as the viral replication resumes using the residual cccDNA as the template^12^. Apparently, eradication of the residual cccDNA is the key for curing HBV infection^7^,^13^. Currently, however, the lack of a convenient technique for producing large quantities of cccDNA limits such cccDNA-orientated studies. Little or no cccDNA is produced in the current *in vitro* experimental systems using plasmids encoding an over-length HBV genome or transgenic mice that harbor an integrated HBV genome^14^,^15^,^16^,^17^.

Most of our understanding on cccDNA biogenesis has been obtained through investigation of duck hepatitis B virus (DHBV)^5^,^18^. Like HBV, DHBV belongs to the hepadnavirus family so that is widely used as a surrogate to study HBV infection, replication, and cccDNA formation^15^,^19^,^20^,^21^. However, DHBV does not equal to HBV. Unlike HBV, for examples, DHBV has a smaller 3-kb genome and does not have the X gene. Given the significant differences in the genome and host between the two viruses, it is reasonable to hypothesize that biology of authentic HBV cccDNA differs significantly from that of DHBV, and a HBV cccDNA model is irreplaceable.

In the present study, we described a protocol based on minicircle technique^22^,^23^ to produce milligrams of recombined cccDNA (rcccDNA) in a common laboratory setting. In addition to demonstrate its function as the wild-type cccDNA, we also showed the CRISPR/Cas9 mediated cleavage of rcccDNA *in vitro* as an example of its application. Thus, the rcccDNA making technique will contribute greatly to the study of cccDNA, such asin searching for the treatment leading to its elimination and the cure of HBV infection.

## Materials and Methods

### Vectors construction and rcccDNA production

**Figure 1:**
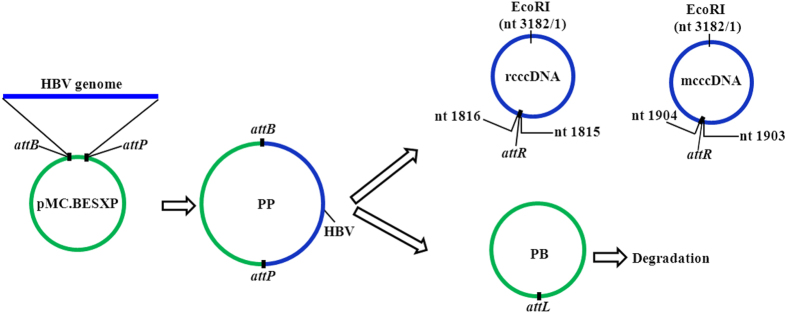
Scheme illustrating production of recombined cccDNAs. The synthesized linear monomeric HBV genomes with different start/end sites (nt 1818-3182/1-1840 for rcccDNA; nt 1904-3182/1-1903 for mcccDNA) were seamlessly sub-cloned into an empty minicircle-producing plasmid (pMC.BESXP), resulting in minicircle-producing plasmid (PP) for rcccDNA and mcccDNA generation, respectively. The PP was used to transform the *E coli*. strain (ZYCY10P3S2T), from which an overnight culture was prepared. L-arabinsoe was added to induce the expression of the DNA recombinase ΦC31 and endonuclease I-SceI integrated in the bacterial genome; the ΦC31 mediated the recombination between the attB and attP built-in in the PP, resulting in the minicircle with a *attR* recombination site, i.e., the rcccDNA or mcccDNA, and the plasmid backbone (PB) DNA circle with a *attL* recombination site, which was linearized by I-SceI and subsequently degraded by bacterial exonucleases. *attB*, bacterial attachment site; *attP*, bacteriophage attachment site; PP, minicircle producing plasmid; PB, plasmid backbone.

Full-length HBV genome sequences based on pTHBV2^24^ with different start/end sites were synthesized by BGI (Shenzhen, China). Using the In-Fusion^®^ HD Cloning Kit (Clontech), the corresponding HBV sequence was sub-cloned into the MC-cloning vector pMC.BESXP^22^ between bacterial attachment *attB* and phage attachment site *attP* to generate parental plasmid (PP) of rcccDNA or mock cccDNA (mcccDNA; Fig 1). After transformed the *E. coli* strain ZYCY10P3S2T^22^ with PP, rcccDNA or mcccDNA were produced, respectively, using the established minicircle DNA preparation process^22^,^23^. The resulted rcccDNA encoded the HBV genome sequence with a 36-bp *attR* recombination site upstream of the preC/C gene, while the mcccDNA has the *attR* site downstream of the ATG initial codon of the preC/C gene.

To construct the plasmids (HBV-gRNA/Cas9) that express the guide RNAs targeting HBV genome sequence and Cas9 nuclease, each pair of HBV-specific-gRNA oligonucleotides listed in Table 1 were annealed and sub-cloned into a sgRNA/Cas9 cloning vector pSpCas9(BB)-2A-GFP (PX458) between the two BbsI restriction sites. The vector pSpCas9(BB)-2A-GFP (PX458) was a gift from Dr. Feng Zhang (Addgene plasmid #48138)^25^.

### Cell culture and transfection

Huh7 cell, purchased from Typical Culture Preservation Commission Cell Bank, Chinese Academy of Sciences (Shanghai, China), was maintained in Dulbecco’s modified Eagle’s medium (DMEM) supplemented with 10% fetal bovine serum (FBS) at 37 °C in a moist atmosphere containing 5% CO_2_. After 24 h of seeding at a density of 10^5^ cells per well of 6-well plates, the cells were transfected with 3 μg rcccDNAs or mcccDNA per well mixed with Lipofectamine 2000 according to the manufacturer’s instructions.

### Southern Blot and Northern Blot analysis

Before Southern or Northern blotting, cytoplasmic viral core particles were isolated from Huh7 cells 48h post-transfection as described previously^26^. The isolated core particles were incubated with 50 U micrococcal Nuclease S7 (Fermentas) to remove the nonencapsidated viral DNA and RNA completely. Core-associated DNA and RNA were extracted from each aliquot of core particles, respectively, as described previously^27^. For Southern blot determination of newly synthesized viral genome, extracted core-associated DNAs were treated with DpnI to remove input rcccDNA. The resulted DNAs and RNAs were separated by agarose gel electrophoresis as described in^27^, and hybridized to a DIG-labeled random-primed probe specific for the HBV sequence. Finally, the viral DNA and RNA bands were illustrated by a horseradish peroxidase-labeled anti-digoxigenin antibody using the DIG-High Prime DNA Labeling and Detection Starter Kit (Roche China, Shanghai).

The digoxigenin (DIG)-labeled probes for Southern and Northern blot were synthesized using the DIG-High Prime DNA Labeling and Detection Starter Kit (Roche), according to the manufacturer’s instructions.

### Detection of viral antigens expression

The levels of HBsAg and HBeAg in the medium of the transfected cells culture were determined periodically by chemiluminiscence using the Abbott ARCHITECT platform (Abbott Laboratories, USA), according to the manufacturer’s instructions.

The expressions of cytoplasmic core-related antigens (HBcAg/HBeAg) in the rcccDNA-transfected Huh7 cells were determined by immunofluorescence analysis. Huh7 cells at 3 days post-transfection were fixed with 4% paraformaldehyde and permeabilized by Triton-X 100. After blockage with 3% bovine serum albumin (BSA) and incubation with (diluted at 1:500 with PBS) rabbit polyclonal anti-HBc antibody (DAKO) for 30 minutes on ice, the cells were incubated with a secondary antibody (Abcam) for 30 minutes at 4 °C. Finally, the cells were counterstained with 4′,6-diamidino-2-phenylindole (DAPI) for 15 minutes at 37 °C, and visualized by a ZEISSLSM710 laser scanning confocal microscope (Carl Zeiss Oberkochen, Germany).

### HBV particles purification

HBV virions were produced from Huh7 cells transfected with rcccDNA, as described previously^28^. Briefly, 3 to 8 days post-transfection, the culture medium was collected, mixed with 6% PEG-8000 (Sigma) and incubated at 4 °C overnight. Subsequently, the mixture was centrifuged at 12,000 *g* at 4 °C for 60 minutes, and the precipitated virions were re-suspended in PBS-10% FBS to a final virion suspension of 10^8^ copies of HBV DNA per ml.

### Chromatin Immune precipitation assays

Chromatin immune precipitation (ChIP) assays were performed as described^29^,^30^. Briefly, 48-hour after transfection with rcccDNA or pTHBV2, Huh-7 cells were fixed in 1% formaldehyde. The cross-linked nuclei were isolated and sonicated in 1% SDS lysis buffer before subjecting to chromatin immunoprecipitation. The reaction was incubated at 4 °C for 14–16 hours after addition of antibodies against H4 (Upstate) and AcH4 (Upstate) and HBcAg (Dako), respectively. Reaction using nonspecific IgG (Abcam) served as negative control. Antibody-DNA-protein complexes were precipitated with protein A/G-conjugated agarose beads and digested with RNase A and proteinase K. The reverse cross-linking was performed at 65 °C for at least 6 hours. DNA was predigested with Plasmid-safe DNase (Epicentre Technologies) before PCR analysis.

### Infection of tree shrew with HBV virion

The animal experiments were performed according to the Guideline for the Care and Use of Laboratory Animals. All animal experimental protocols were approved by the Ethics committee of the Animal Laboratory of Shenzhen Institutes of Advanced Technology, Chinese Academy of Sciences (Permit Number: SIAT-IRB-120830-A0058).

The infectious ability of the rcccDNA-produced virions was determined using a tree shrew model. Female tree shrews (Tupaia, *belangeri chinensis*) obtained from the Kunming Institute of Zoology, Chinese Academy of Science (Kunming, China) were housed under standard pathogen-free (SPF) conditions at the Laboratory Animal Center of Jinan University (Guangzhou, China). The tree shrews (n = 21) were divided into three groups randomly: the rcccDNA-derived virion (n = 11) group, blank control (n = 5) group, and patient’s HBV (n = 5) group. Before virion inoculation (day 0), blood sample was collected from each animal to serve as a baseline and to exclude pre-existing HBV infection. Each tree shrew in the rcccDNA-derived virion group was inoculated with 1 ml purified viral particles from rcccDNA-transfected huh7 cell culture, the viral particles were re-suspended in PBS-10% FBS (10^8^ copies/ml), whereas those in the blank control group were inoculated with PBS containing 10% FBS (1 ml per tree shrew). For patient’s HBV group, each animal was inoculated with 1 ml human serum (10^8^ copies of HBV DNA genome per ml) obtained from a CHB patient in the Third People’s Hospital of Shenzhen, China. All experimental protocol, including collection and use of HBV patient’s serum, was approved by the Ethics Committee of Shenzhen Third People’s Hospital with a written consent from the patient. The protocol was conducted in accordance with the Declaration of Helsinki.

Blood sample was collected from each tree shrew 4, 9, 15, and 28 days post-inoculation. The expression of serum HBV biomarkers, including HBV surface antigen (HBsAg), HBsAb (HBV surface antibody), HBV e-antigen (HBeAg), HBV e-antibody (HBeAb), HBV core antibody (HBcAb), and viral DNA were determined periodically.

### Immunohistochemistry and histological assays of the liver tissue

The tree shrew livers were biopsied 9 days after inoculation. A portion of each liver biopsy was fixed in 10% (v/v) neutral formalin and then embedded in paraffin for routine histological and immunohistochemistry examinations. The expression of HBcAg in the paraffin-embedded liver biopsies was examined by Immunohistochemistry assays using the monoclonal antibody of mouse anti-human HBcAg (Maxim Bio, Fuzhou, China) and UltraSensitive^TM^ S-P staining kit (Maxim Bio, Fuzhou, China) and DAB kit (Maixin Bio, Fuzhou, China), according to the manufacturers’ instructions. To better localize the HBV infected cells, the liver antibody-stained samples were co-stained with hematoxylin and eosin (HE)-staining.

### Detection of cccDNA by PCR

The cccDNA was extracted from the tree shrew livers at the end of the 28-day experiments using the procedure described by Guo *et al*.^14^ with modification. Using the extracted DNA as template, a PCR-based method described previously^31^ was used to the detection of cccDNA. An elongation step using a chimeric primer (5′-TCGCTTTCGGGTCCCTCATGCGACGTGC-3′), which is capable of selectively generating elongation product from cccDNA, but not rcDNA, to serve as qPCR template before PCR reaction to distinguish the cccDNA from rcDNA. The qPCR primers for cccDNA detection are cccDNA.fluo.P1(F) (5′-TCGCTTTCGGGTCCCT-3′) and cccDNA.fluo.P2(Rev) (5′-GCACCTCTCTTTACGCGGTC-3′), and a taqman probe (5′-CCCGTCTGTGCCTTCTCATCTGCCG-3′) was used in the PCR system to report the detection signal.

### Determination of Crispr/Cas9-mediated cccDNA clearance

Huh7 were co-transfected with rcccDNA (or mcccDNA) and HBV-gRNA/Cas9 dual expression plasmid. The level of secretory viral antigens (HBsAg and HBeAg), pre-genomic RNA (pgRNA) and cccDNA were detected at 48 h post-transfection. The secretory antigens and new-synthesized (namely cell-born) cccDNA were quantified by chemiluminiscence and qPCR as described above, respectively. While the pgRNA was quantified by quantitative real-time PCR. In brief, the extracted total cellular RNA was reverse-transcribed into cDNA using PrimeScript™ RT-PCR Kit (Takara, Japan) according to the manufacturer’s instructions. The cDNA samples were subjected to real-time PCR using the following pgRNA specific primers: pgRNA-F (5′-GCCTTAGAGTCTCCTGAGCA-3′), pgRNA-R (5′-GAGGGAGTTCTTCTTCTAGG-3′), where the quantitative real-time PCR was performed on the Cobas^®^TaqMan^®^ real time–polymerase chain reaction analyzer (Roche Diagnostics, USA). The relative amount of pgRNA in each sample was normalized to that of β-actin.

T7E1 assay^32^ was conducted to investigate if HBV-gRNA/Cas9 is able to interrupt pre-existing cccDNA. The huh7 cells were co-transfected with mcccDNA and HBV-gRNA/Cas9 dual expression plasmid. After 48 hours culture, the nuclear DNA from the transfected huh7 cells were isolated following the method described by Cohen *et al*.^33^. The DNA fragment covering the HBV-sgRNA target site was amplified using either the rcccDNA or the isolated nuclear DNA from mcccDNA-transfected cells as the template, respectively. Then the PCR products was mixed (1:1), melted and annealed. The annealed DNA was treated with 5 units of T7 endonuclease 1 (T7E1, NEB), which cuts the heteroduplexes but not the homoduplexes, for 20 min at 37 °C before analyzed by agarose gel electrophoresis^34^.

### Statistical Analysis

Statistical analysis was performed using Students’ t-test and results with a *P*-value less than 0.05 were considered significant.

## Results

### Production of HBV rcccDNA using minicircle technology

rcccDNA was generated from a minicircle-producing plasmid harboring a 3.2 kb HBV monomeric genome DNA using a ΦC31-mediated DNA recombination for the minicircle production^22^,^23^. Milligrams of rcccDNA was produced from 400-ml of overnight culture with the purity of up to 99.7% using the enhanced technology^23^. Within the minicircle-producing plasmid, the monomeric (one unit) HBV genome sequence was placed between the bacterial attachment site (attB) and phage attachment site (attP). Upon recombination, a circular DNA encoding the monomeric HBV genome together with a 36-bp attR recombination site was produced. Because the 36-bp attR residual site can disrupts the highly compact HBV genome, the location of this recombination site was carefully designed. To avoid disruption of viral genome, in terms of coding genes and regulatory elements, we placed the attR site into the upstream of preC/C ORF (nt. 1816). The coding region of preC/C is from 1816 to 2454, while the X ORF is from 1376 to 1840; it is obvious that there is a 25-bp narrow overlapped region (nt. 1816–1840) between these two ORFs. We added an additional HBV sequence (nt.1816–1840 that contains a stop coden) before the preC/C (nt 1816) to guarantee the expression of a full-length X protein. Consequently, a 3.2 kb rcccDNA containing monomeric HBV genome and a 25 bp redundant sequence (nt. 1816–1840) with a 36-bp attR recombination site was produced (Fig. 1). To serve as a nonfunctional negative control, we produced another mcccDNA comprising monomeric HBV genome with the attR located in the N-terminal of the C gene (nt. 1904); this resulted in a stop codon immediately following the ATG initiation codon of the C gene. Theoretically, the mcccDNA will produce no core protein and, consequently, no infectious HBV virions as determined in a huh7 cell transfection study (data not shown).

### rcccDNA-mediated HBV replication in Huh7 cells

**Figure 2:**
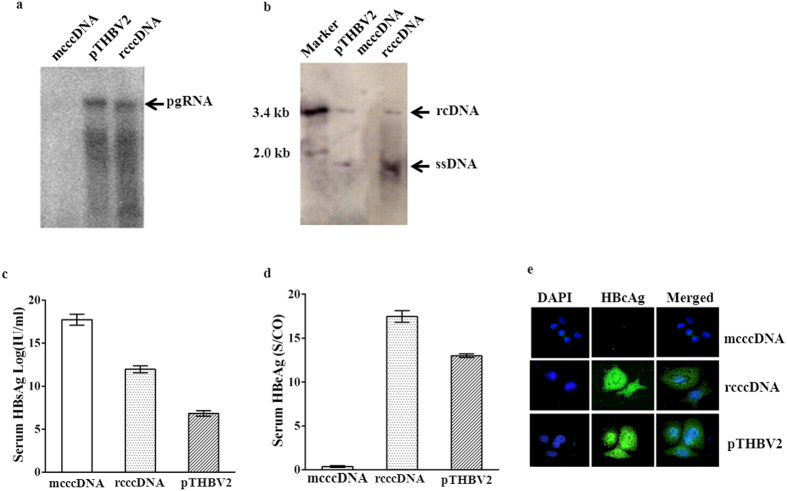
rcccDNA mediated HBV expression and replication *in vitro*. Cultured Huh7 cells were transfected with either rcccDNA, mcccDNA or pTHBV2 DNA using Lipofectamine. The cells were harvested 48- or 72 h post transfection and processed for analysis of viral products. The core-associated viral RNA or DNA was detected by Northern blot (**a**) or Southern blot (**b**) in Huh7 cells 48 h after transfection of rcccDNA or pTHBV2. The levels of circulating HBsAg (**c**) or HBeAg (**d**) 72 h post-transfection were determined by Chemiluminiscence. Intracellular HBcAg 72 h post-transfection was illustrated by immune-staining followed by confocal microscopy (**e**).

In the natural HBV life cycle, the cccDNA serves as the transcriptional template for synthesizing viral RNAs; among them, the pre-genomic RNA (pgRNA) is encapsidated by the viral core protein to form the capsid and subsequently served as a template to synthesize the viral DNA genome. To verify the transcriptional function of our rcccDNA, we transfected the huh7 with the rcccDNA and mcccDNA, respectively, and harvested the cells 48 hours post-transfection to analyze its pgRNA using Northern Blot technique. As a positive control, wild-type pgRNA was prepared from pTHBV2-transfected Huh7 cells. The expected pgRNA signal was detected from both rcccDNA and pTHBV2-transfected groups, but not in the mcccDNA group (Fig. 2a). The absence of viral RNA in the mcccDNA group is because the experiment was designed to detect the core-particle associated RNA which would be lacking in the group. It is very possible that the insert of the *attR at the* N-terminal of PreC/C ORF had disabled the producing of core particle.

To further evaluate the replication competency of rcccDNA, Southern Blot was conducted to determine the viral DNA within capsids at the same time point as Northern Blot. As shown in Fig. 2b, the replicative intermediates (rcDNA and single-stranded DNA (ssDNA)) were detected in both the rcccDNA and pHBV2 groups, but not the mcccDNA group.

We found that rcccDNA produced comparable amount of core-associated -rcDNA (rcccDNA/pTHBV2 = 0.96) or –pgRNA (rcccDNA/pTHBV2 = 1.08) with the wild-type counterpart (pTHBV2) in Huh7 cell. Where the relative quantification of rcDNA and pgRNA was performed using software Image J based on estimating the gray scale of their corresponding bands (the density of band).

Both HBsAg (Fig. 2c) and HBeAg (Fig. 2d) signals were much stronger in the rcccDNA-transfected Huh7 cell than in the pTHBV2 plasmid-transfected cells 72 hours after transfection. Furthermore, HBcAg was detected in both the cytoplasm and nucleus, reflecting a nucleo-cytoplasmic translocating feature of HBcAg (Fig. 2e).

These data clearly demonstrated the existence of rcccDNA-mediated HBV replication and viral gene expression in Huh7 cells.

### rcccDNA is organized as cccDNA-like minichromosome

**Figure 3:**
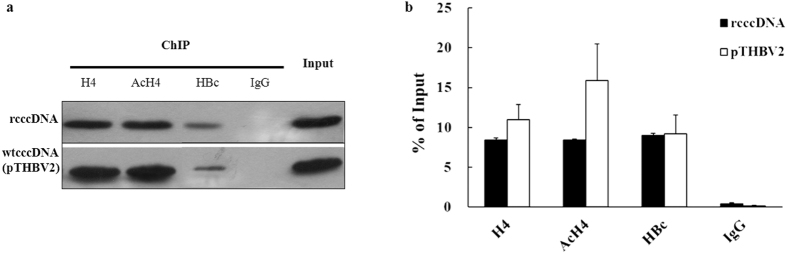
rcccDNA is organized as a minichromosome in hepatoma cell line. (**a**) ChIP of Huh-7 cells transfected with rcccDNA (or pTHBV2 plasmid) using antibodies against H4, AcH, HBcAg and IgG (negative control). (**b**) Quantitation of immunoprecipitated rcccDNA by real-time PCR. Data (mean ± SD) were expressed as percentage of input.

Wild-type cccDNA (wtcccDNA) is epigenetically organized as minichromosome in the hepatocytes. To investigate if the MC-based rcccDNA can also form such a minichromosome, we designed a ChIP assay to study this feature. Like wtcccDNA derived from the plasmid pTHBV2 encoding oversize HBV genome, rcccDNA was associated with histone H4 and its acetylated form (AcH4), and the nonhistone HBcAg as well (Fig. 3a). The quantity of the immunoprecipitated rcccDNA was determined by real-time PCR using HBV-specific primers (Fig. 3b). Taking together, these data showed that rcccDNA was epigenetically organized as a minichromosome similar to the wild-type HBV cccDNA.

### Infectivity of rcccDNA-derived HBV virion in tree shrews

**Figure 4:**
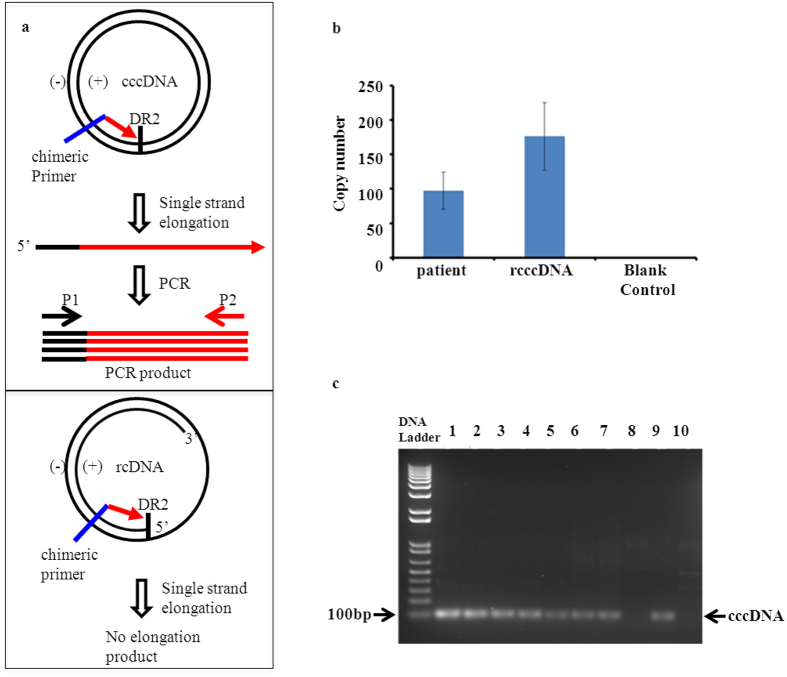
Detection of cccDNA in tree shrew liver samples. Total liver DNA was prepared from tree shrew liver samples to serve as PCR template. (**a**) Scheme illustrating the principle of rcccDNA detection by PCR. The chimeric primer, which served as elongation primer, comprised two parts: the 3′ half is complementary to the sequence before the recombination point at direct repeat 2 (DR2) so that is capable of producing the elongation product from the cccDNA template, but not from the rcDNA; the 5′ part, non-homogenies with HBV genome, encodes a PCR primer (P1), which is used, in couple with another primer (P2), to generate PCR product using the elongation product as template. (**b**) Quantitation of copy number of cccDNA by qPCR. Mean ± SD; rcccDNA group, n = 7; patient group, n = 5; blank control group, n = 5; (**c**) Detection of cccDNA by conventional PCR. Lane 1–7: rcccDNA group, gradually diluted (10 x) liver DNAs; Lane 8: non-single strand elongation control of rcccDNA group; Lane 9: patient’s group; Lane 10: non-single strand elongation control of patient’s group.

In addition to transcription and replication, ability in production of infectious virions is another critical function of HBV cccDNA in nature. To this end, we isolate the virions from culture medium of rcccDNA-transfected Huh7 cells and test their infectivity using a tree shrew model. Of the 11 tree shrews inoculated with the rcccDNA-derived virus, 7 were diagnosed with HBV infection. Specifically, serum HBsAg, HBV DNA and HBcAb were detected in the blood samples of these infected animals 4 days after virion inoculation, while the antibody against HBsAg (HBsAb) appeared 15 days post-inoculation. However, HBeAg and HBeAb were absent throughout the experiment (Table 2). The HBsAg and viral DNA disappeared from the serums of all the infected animals within 15 days post-inoculation, while the HBV-specific antibodies (HBsAb and HBcAb) remained positive at the end of experiment (28 days post-inoculation), suggesting a process of spontaneous clearance of HBV after the establishment of an acute infection in these tree shrews. A similar infection-recovery process was seen in all the 5 animals injected with HBV virus-positive patient’s blood. The only difference was that either HBeAg or HBeAb was transiently detectable in the serum in this group. Interestingly, using a PCR-based methodology^31^ (see Schematic Fig. 4a), both real-time fluorescence (Fig. 4b) and convenient PCR (Fig. 4c) showed that rcccDNA, with the specific marker *attR*, remained in the infected tree shrew liver tissue 28 days after inoculation, when almost all of the serum HBV-specific markers had disappeared. Although the duration of the rcccDNA persists in the liver remains to be determined, this result is reminiscent of the observations that trace amount of HBV cccDNA is detectable in human liver^35^, and that immune suppression, cancer chemotherapy and steroid treatment are able to trigger viral reactivation and hepatitis flare^36^ long after the HBV infection had been resolved. It is possible that our rcccDNA can be used to unravel the mechanism leading to viral silencing and reactivation.

**Figure 5:**
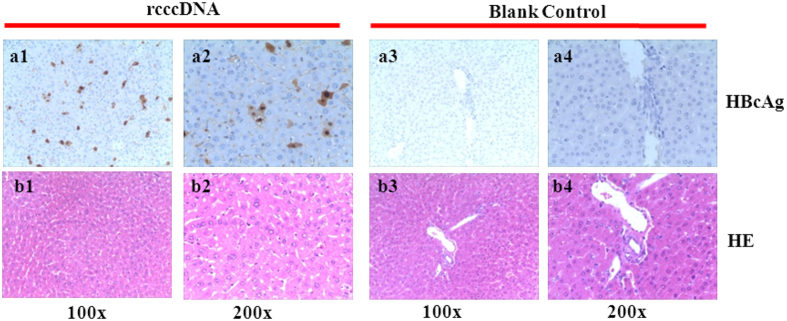
HBcAg in tree shrew livers illustrated by immunohistochemistry. HBcAg in the rcccDNA-derived virion group (upper row-left), and the blank control group (upper row-right; magnification 100× and 200×); Histopathological view of the tree shrew livers of the rcccDNA- (lower row-left) and patient group (lower row-right, HE. staining).

Immunohistochemical staining for the liver biopsy of the tree shrews inoculated with rcccDNA-derived virus (resuspended in PBS-10% FBS) demonstrated a sporadic pattern of HBcAg-positive hepatocytes manifested as yellowish-brown granules (Fig. 5a, left); however, the liver biopsy from the blank control (PBS with 10% PBS) was negative for HBcAg staining (Fig. 5a, right). Unexpectedly, in contrast to the positive biochemistry and histology findings of viral biomarkers, no liver injury was seen at the histology level in the rcccDNA group (Fig. 5b).

### Cleaving cccDNA using Crispr/Cas9 system

To investigate the potential of gRNA-Cas9 in cleaving the cccDNA and serving as a therapeutic means, we co-transfected Huh7 cells with 1 μg rcccDNA and HBV-gRNA/Cas9 expressing plasmid at a molar ratio of 1:1. As a blank control, the PX458 plasmid expressing the Cas9 without the gRNA was used in place of the gRNA/Cas9 expressing plasmid.

**Figure 6:**
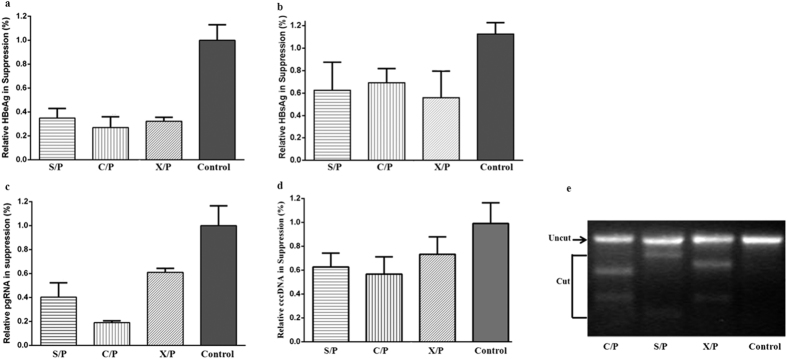
Crispr/Cas9 mediate HBV disruption. Huh7 cells were co-transfected with the rcccDNA and HBV-gRNA/Cas9 expression plasmids. After 48 h of transfection, the supernatants were collected to detect HBeAg (**a**) and HBsAg (**b**) by chemiluminiscence using the Abbott ARCHITECT platform, pgRNAs (**c**) and new-synthesized cell-born cccDNA (**d**) were quantified by real-time PCR analysis. The nuclear MC, isolated from the mcccDNA and HBV-gRNA/Cas9 expression plasmids co-transfected Huh7 cells, that can surrrogate the pre-existing cccDNA was subjected to a T7E1 assay to detect Crispr/Cas9-induced cccDNA disruption (**e**).

As shown in Fig. 6, all the three designed gRNA-Cas9 plasmids were able to significantly reduce the expression of HBeAg (*p* < 0.01; Fig. 6a) and HBsAg (*p* < 0.05; Fig. 6b) as determined by the chemiluminiscence method. As expected, the quantity of pgRNA in gRNAs treated groups was significantly reduced (*p* < 0.01) as compared to that of the control (Fig. 6c) 48 hours post-transfection as determined by real-time PCR. Furthermore, the amounts of the cell-born cccDNA in huh7 cells was dramatically decreased in gRNAs-expressing cells (Fig. 6d). Nevertheless, the decrease of cccDNA molecule did not guarantee the HBV-gRNA/Cas9 mediated cleavage of pre-existing cccDNA. To this end, we performed a T7E1 assay to clarify this question. As shown in Fig. 6e, amplified DNA fractions from mcccDNA and gRNA-Cas9 treated cells were cleaved by T7E1, indicating that HBV-specific sgRNAs did cut the target sites of HBV genome.

## Discussion

In this study, we have developed a minicircle-based technique^22^,^23^ to produce milligrams of HBV rcccDNA in common molecular biology laboratory setting. The minicircle-based rcccDNA closely resembles to its wild-type counterpart in structure and function. It encodes one unit of the HBV genome with a 36-bp recombination hybrid *attR*. Importantly, we have demonstrated that this rcccDNA mimicked wild-type cccDNA in functions closely, including the expression of all the viral RNAs and proteins. Furthermore, the virions derived from the rcccDNA were able to infect tree shrews, and to produce viral products in the circulation and livers, as determined by PCR, ELISA, and immunohistochemistry. Thus, the minicircle-based rcccDNA can be used as an HBV cccDNA surrogate in the study of HBV biology and in the development of anti-cccDNA drugs, which are needed badly to terminate chronic HBV infection and to halt the progress to cirrhosis and liver cancer that threaten millions of people worldwide.

It is very easy to produce rcccDNA by using the minicircle technology comprising pMC.BESXP vector and ZYCY10P3T2S *E. coli*^22^,^37^. The only requirements are to place the full-length HBV genome into the minicircle-producing plasmid and to design the *attR* in an appropriate location. The production procedure is similar to that for other minicircles^22^. We made two minicircles, each encoded one unit of HBV genome, and only the one with the *attR* locating upstream of the start codon of the preC/C gene worked. We name this minicircle as rcccDNA because it was able to function as the wtcccDNA as described above. The other one, named as mcccDNA, with the *attR* at the N-terminal of PreC/C gene (downstream of the start codon) failed to work, probably due to the malfunctioning of the core protein which was altered by the insert of extra 12 amino acids derived from the 36-bp *attR* right after the start codon of the PreC/C gene.

During the past decade, a few *in vitro* cccDNA-producing systems have been established. For example, Günther and colleagues developed a PCR-enzymatic ligation based method to make rcccDNA^38^. However, the procedure is labor-intensive and the yield is low. Recently, Qi *et al*.^30^ reported an HBV cccDNA surrogate based on a Cre-loxp-mediated site-specific DNA recombination. This technique, although useful, has multiple shortcomings, including (1) the cccDNA exits only in the cell or mouse liver, and is not available in test tube for additional applications; (2) unlike ΦC31, which mediates a unidirectional recombination reaction so that capable of producing a unique monomer population comprising one unit of HBV genome as the wtcccDNA, the Cre-Loxp system directs a bidirectional reaction, resulting in multiple populations of DNA circles, including HBV monomer, dimer, multimer, and HBV genome-plasmid backbone heterogeneous populations^39^,^40^. The third example is the Bac-HBV system in which the recombinant baculovirus encoding an over-length HBV genome is able to generate cccDNA in hepatoma cell^41^,^42^. However, the baculovirus particle preparation is a time- and labor-consuming process which limit the application of the system. Additional technologies, such as cell lines expressing HBV receptor, including NTCP-HepG2^43^,^44^ or NTCP-Huh7^44^, can also produce detectable cccDNA upon infection with HBV virion. However, establishment of HBV infection in these cell lines is hard because it requires a huge excess of virus particles--up to 10^4^-fold of the cells^44^. Taken together, our minicircle-based cccDNA model is a better one because it is convenient to make milligrams of high quality rcccDNA with full function of wtcccDNA.

In summary, it is reasonable to expect that the minicircle-based rcccDNA will play an important role in the study of HBV biology. For example, it will be easy to make a HBV chronic infection mouse model by hydrodynamic injecting rcccDNA into mouse, due to the rcccDNA-mediated viral lasting expression (Fig. S1)^45^,^46^. This *in vivo* HBV model will overmatch either the HBV-plasmid based mouse model where the silencing effect of plasmid backbone limits the viral gene expression duration^37^ or the HBV genome integrated mouse model where it lacks cccDNA formation for an unknown reason^30^. It is worth of highlighting the application of rcccDNA for searching for anti-cccDNA agents. In the present study, we have already successfully used the MC-based rcccDNA to confirm the efficient cleavage of cccDNA by gRNA/Cas9. Our results have univocally demonstrated the wide application value of our minicircle-based rcccDNA-making technology.

## Additional Information

**How to cite this article**: Guo, X. *et al*. The recombined cccDNA produced using minicircle technology mimicked HBV genome in structure and function closely. *Sci. Rep.* **6**, 25552; doi: 10.1038/srep25552 (2016).

## Supplementary Material

Supplementary Information

## Figures and Tables

**Table 1:** The gRNAs targeted-sequence located in the HBV genome.

**Name**	**gRNA (GN**_**19/20**_**NGG)**	**Target Sequence**	**Nucleotide Position**
gRNA-S/P	gCTGAGGCCCACTCCCATAGG	CCTATGGGAGTGGGCCTCAG	637–656
gRNA-X/P	GTGAAGCGAAGTGCACACGG	CCGTGTGCACTTCGCTTCAC	1575–1594
gRNA-C/P	gTGAGATCTTCTGCGACGCGG	CCGCGTCGCAGAAGATCTCA	2453–2472

The NGG protospacer adjacent motif (PAM) was underlined.

**Table 2:** Detection of the serum HBV-specific markers after inoculation of rcccDNA-derived virus in tree shrews.

**Indicators**	**Time Points**	**Blank control (n = 5)**					**rcccDNA (n = 11)**											**HBV patients (n = 5)**				
		**1**	**2**	**3**	**4**	**5**	**6**	**7**	**8**	**9**	**10**	**11**	**12**	**13**	**14**	**15**	**16**	**17**	**18**	**19**	**20**	**21**
HBsAg	d0	**−**	**−**	**−**	**−**	**−**	**−**	**−**	**−**	**−**	**−**	**−**	**−**	**−**	**−**	**−**	**−**	**−**	**−**	**−**	**−**	**−**
	d4	**−**	**−**	**−**	**−**	**−**	**+**	**+**	**−**	**−**	**+**	**+**	**+**	**+**	**−**	**−**	**−**	**+**	**+**	**+**	**+**	**+**
	d9	**−**	**−**	**−**	**−**	**−**	**+**	**+**	**−**	**−**	**+**	**+**	**+**	**+**	**−**	**−**	**−**	**+**	**+**	**+**	**+**	**+**
	d15	**−**	**−**	**−**	**−**	**−**	**−**	**−**	**−**	**−**	**−**	**−**	**−**	**−**	**−**	**−**	**−**	**+**	**+**	**+**	**+**	**+**
	d28	**−**	**−**	**−**	**−**	**−**	**−**	**−**	**−**	**−**	**−**	**−**	**−**	**−**	**−**	**−**	**−**	**−**	**−**	**−**	**−**	**−**
HBsAb	d0	**−**	**−**	**−**	**−**	**−**	**−**	**−**	**−**	**−**	**−**	**−**	**−**	**−**	**−**	**−**	**−**	**−**	**−**	**−**	**−**	**−**
	d4	**−**	**−**	**−**	**−**	**−**	**−**	**−**	**−**	**−**	**−**	**−**	**−**	**−**	**−**	**−**	**−**	**−**	**−**	**−**	**−**	**−**
	d9	**−**	**−**	**−**	**−**	**−**	**−**	**−**	**−**	**+**	**−**	**−**	**−**	**−**	**−**	**−**	**−**	**−**	**−**	**−**	**−**	**−**
	d15	**−**	**−**	**−**	**−**	**−**	**+**	**+**	**−**	**+**	**+**	**+**	**+**	**+**	**−**	**−**	**−**	**−**	**−**	**−**	**−**	**−**
	d28	**−**	**−**	**−**	**−**	**−**	**+**	**+**	**−**	**+**	**+**	**+**	**+**	**+**	**−**	**−**	**−**	**+**	**+**	**+**	**+**	**+**
HBeAg	d0	**−**	**−**	**−**	**−**	**−**	**−**	**−**	**−**	**−**	**−**	**−**	**−**	**−**	**−**	**−**	**−**	**−**	**−**	**−**	**−**	**−**
	d4	**−**	**−**	**−**	**−**	**−**	**−**	**−**	**−**	**−**	**−**	**−**	**−**	**−**	**−**	**−**	**−**	**+**	**+**	**+**	**−**	**−**
	d9	**−**	**−**	**−**	**−**	**−**	**−**	**−**	**−**	**−**	**−**	**−**	**−**	**−**	**−**	**−**	**−**	**+**	**+**	**+**	**−**	**−**
	d15	**−**	**−**	**−**	**−**	**−**	**−**	**−**	**−**	**−**	**−**	**−**	**−**	**−**	**−**	**−**	**−**	**+**	**+**	**+**	**−**	**−**
	d28	**−**	**−**	**−**	**−**	**−**	**−**	**−**	**−**	**−**	**−**	**−**	**−**	**−**	**−**	**−**	**−**	**−**	**−**	**−**	**−**	**−**
HBeAb	d0	**−**	**−**	**−**	**−**	**−**	**−**	**−**	**−**	**−**	**−**	**−**	**−**	**−**	**−**	**−**	**−**	**−**	**−**	**−**	**−**	**−**
	d4	**−**	**−**	**−**	**−**	**−**	**−**	**−**	**−**	**−**	**−**	**−**	**−**	**−**	**−**	**−**	**−**	**−**	**−**	**−**	**+**	**+**
	d9	**−**	**−**	**−**	**−**	**−**	**−**	**−**	**−**	**−**	**−**	**−**	**−**	**−**	**−**	**−**	**−**	**−**	**−**	**−**	**+**	**+**
	d15	**−**	**−**	**−**	**−**	**−**	**−**	**−**	**−**	**−**	**−**	**−**	**−**	**−**	**−**	**−**	**−**	**−**	**−**	**−**	**+**	**+**
	d28	**−**	**−**	**−**	**−**	**−**	**−**	**−**	**−**	**−**	**−**	**−**	**−**	**−**	**−**	**−**	**−**	**−**	**−**	**−**	**−**	**−**
HBcAb	d0	**−**	**−**	**−**	**−**	**−**	**−**	**−**	**−**	**−**	**−**	**−**	**−**	**−**	**−**	**−**	**−**	**−**	**−**	**−**	**−**	**−**
	d4	**−**	**−**	**−**	**−**	**−**	**+**	**+**	**−**	**−**	**+**	**+**	**+**	**+**	**−**	**−**	**−**	**+**	**+**	**+**	**+**	**+**
	d9	**−**	**−**	**−**	**−**	**−**	**+**	**+**	**−**	**+**	**+**	**+**	**+**	**+**	**−**	**−**	**−**	**+**	**+**	**+**	**+**	**+**
	d15	**−**	**−**	**−**	**−**	**−**	**+**	**+**	**−**	**+**	**+**	**+**	**+**	**+**	**−**	**−**	**−**	**+**	**+**	**+**	**+**	**+**
	d28	**−**	**−**	**−**	**−**	**−**	**+**	**+**	**−**	**+**	**+**	**+**	**+**	**+**	**−**	**−**	**−**	**+**	**+**	**+**	**+**	**+**
HBV DNA	d0	**−**	**−**	**−**	**−**	**−**	**−**	**−**	**−**	**−**	**−**	**−**	**−**	**−**	**−**	**−**	**−**	**−**	**−**	**−**	**−**	**−**
	d4	**−**	**−**	**−**	**−**	**−**	**+**	**+**	**−**	**−**	**+**	**+**	**+**	**+**	**−**	**−**	**−**	**+**	**+**	**+**	**+**	**+**
	d9	**−**	**−**	**−**	**−**	**−**	**+**	**+**	**−**	**−**	**+**	**+**	**+**	**+**	**−**	**−**	**−**	**+**	**+**	**+**	**+**	**+**
	d15	**−**	**−**	**−**	**−**	**−**	**−**	**−**	**−**	**−**	**−**	**−**	**−**	**−**	**−**	**−**	**−**	**+**	**+**	**+**	**−**	**−**
	d28	**−**	**−**	**−**	**−**	**−**	**−**	**−**	**−**	**−**	**−**	**−**	**−**	**−**	**−**	**−**	**−**	**−**	**−**	**−**	**−**	**−**
infected		N	N	N	N	N	Y	Y	N	Y	Y	Y	Y	Y	N	N	N	Y	Y	Y	Y	Y

HBV-specific markers, including serum HBsAg, HBsAb, HBeAg, HBeAb, HBcAb, and viral DNA were tested in tree shrew serum at distinct time points (0, 4, 9, 15, and 28 days post-injection). The minus sign (−) represents a negative result; the plus sign (+) represents a positive result; Y or N indicates that the corresponding tree shrew had or had not HBV infection, respectively.
