# Supplementary material for: The recombined cccDNA produced using minicircle technology mimicked HBV genome in structure and function closely
Source: Sci Rep. 2016 May 13;6:25552. doi: 10.1038/srep25552 (PMC4865889; doi:10.1038/srep25552)
Supplement: Supplementary Information [file srep25552-s1.pdf]

The recombined cccDNA produced using minicircle technology mimicked HBV genome in structure and function closely

Xiaoyan Guo,[1,2†] Ping Chen,[1†*] Xiaohu Hou,[1] Wenjuan Xu,[1] Dan Wang,[1]Tian-yan Wang,[1] Gang Zheng,[1] Zhi-liang Gao,[2] Cheng-Yi He,[1] Boping Zhou,[3*] and Zhi-Ying Chen[1*]

1, The Laboratory for Gene and Cell Engineering, Shenzhen Institutes of Advanced Technology, Chinese Academy of Sciences, Shenzhen, 518055, China.

2, Department of Infectious Diseases, The Third Affiliated Hospital of Sun Yat-Sen University, 600 Tian He Road, Guangzhou, 510630, China.

3, Institute of Hepatology, Shenzhen Third People's Hospital, Shenzhen, 518112, China.

† The authors contributed equally to this work

* Corresponding authors

Zhi-Ying Chen, E-mail: zy.chen1@siat.ac.cn;

Boping Zhou, E-mail: zhoubp@hotmail.com;

Ping Chen, E-mail: chenping@siat.ac.cn

S1 rcccDNA sequence

ATGCAACTTTTTCACCTCTGCCTAATCATCTCTTGTTCATGTCCTACTGTTCAAGCCTCCAAGCTGTGCCTTGGGTGG

DR2 epsilon

CTTTGGGGCATGGACATCGACCCTTATAAAGAATTTGGAGCTACTGTGGAGTTACTCTCGTTTTTGCCTTCTGACTT

poly A

CTTTCCTTCAGTACGAGATCTTCTAGATACCGCCTCAGCTCTGTATCGGGAAGCCTTAGAGTCTCCTGAGCATTGTT
CACCTCACCATACTGCACTCAGGCAAGCAATTCTTTGCTGGGGGGAACTAATGACTCTAGCTACCTGGGTGGGTG
TTAATTTGGAAGATCCAGCGTCTAGAGACCTAGTAGTCAGTTATGTCAACACTAATATGGGCCTAAAGTTCAGGCA
ACTCTTGTGGTTTCACATTTCTTGTCTCACTTTTGGAAGAGAAACAGTTATAGAGTATTTGGTGTCTTTCGGAGTGT
GGATTCGCACTCCTCCAGCTTATAGACCACCAAATGCCCCTATCCTATCAACACTTCCGGAGACTACTGTTGTTAGA
CGACGAGGCAGGTCCCCTAGAAGAAGAACTCCCTCGCCTCGCAGACGAAGGTCTCAATCGCCGCGTCGCAGAAG
ATCTCAATCTCGGGAATCTCAATGTTAGTATTCCTTGGACTCATAAGGTGGGGAACTTTACTGGGCTTTATTCTTCTA
CTGTACCTGTCTTTAATCCTCATTGGAAAACACCATCTTTTCCTAATATACATTTACACCAAGACATTATCAAAAAATG
TGAACAGTTTGTAGGCCCACTCACAGTTAATGAGAAAAGAAGATTGCAATTGATTATGCCTGCCAGGTTTTATCCA
AAGGTTACCAAATATTTACCATTGGATAAGGGTATTAAACCTTATTATCCAGAACATCTAGTTAATCATTACTTCCAAA
CTAGACACTATTTACACACTCTATGGAAGGCGGGTATATTATATAAGAGAGAAACAACACATAGCGCCTCATTTTGT
GGGTCACCATATTCTTGGGAACAAGATCTACAGCATGGGGCAGAATCTTTCCACCAGCAATCCTCTGGGATTCTTT
CCCGACCACCAGTTGGATCCAGCCTTCAGAGCAAACACCGCAAATCCAGATTGGGACTTCAATCCCAACAAGGAC
ACCTGGCCAGACGCCAACAAGGTAGGAGCTGGAGCATTCGGGCTGGGTTTCACCCCACCGCACGGAGGCCTTTT
GGGGTGGAGCCCTCAGGCTCAGGGCATACTACAAACTTTGCCAGCAAATCCGCCTCCTGCCTCCACCAATCGCCA
GTCAGGAAGGCAGCCTACCCCGCTGTCTCCACCTTTGAGAAACACTCATCCTCAGGCCATGCAGTGGAATTCCAC
AACCTTCCACCAAACTCTGCAAGATCCCAGAGTGAGAGGCCTGTATTTCCCTGCTGGTGGCTCCAGTTCAGGAAC
AGTAAACCCTGTTCTGACTACTGCCTCTCCCTTATCGTCAATCTTCTCGAGGATTGGGGACCCTGCGCTGAACATGG
AGAACATCACATCAGGATTCCTAGGACCCCTTCTCGTGTTACAGGCGGGGTTTTTCTTGTTGACAAGAATCCTCAC
AATACCGCAGAGTCTAGACTCGTGGTGGACTTCTCTCAATTTTCTAGGGGGAACTACCGTGTGTCTTGGCCAAAAT
TCGCAGTCCCCAACCTCCAATCACTCACCAACCTCTTGTCCTCCAACTTGTCCTGGTTATCGCTGGATGTGTCTGCG
GCGTTTTATCATCTTCCTCTTCATCCTGCTGCTATGCCTCATCTTCTTGTTGGTTCTTCTGGACTATCAAGGTATGTTG
CCCGTTTGTCCTCTAATTCCAGGATCCTCAACAACCAGCACGGGACCATGCCGGACCTGCATGACTACTGCTCAAG
GAACCTCTATGTATCCCTCCTGTTGCTGTACCAAACCTTCGGACGGAAATTGCACCTGTATTCCCATCCCATCATCCT
GGGCTTTCGGAAAATTCCTATGGGAGTGGGCCTCAGCCCGTTTCTCCTGGCTCAGTTTACTAGTGCCATTTGTTCA
GTGGTTCGTAGGGCTTTCCCCCACTGTTTGGCTTTCAGTTATATGGATGATGTGGTATTGGGGGCCAAGTCTGTAC
AGCATCTTGAGTCCCTTTTTACCGCTGTTACCAATTTTCTTTTGTCTTTGGGTATACATTTAAACCCTAACAAAACAA
AGAGATGGGGTTACTCTCTAAATTTTATGGGTTATGTCATTGGATGTTATGGGTCCTTGCCACAAGAACACATCATA
CAAAAAATCAAAGAATGTTTTAGAAAACTTCCTATTAACAGGCCTATTGATTGGAAAGTATGTCAACGAATTGTGG
GTCTTTTGGGTTTTGCTGCCCCTTTTACACAATGTGGTTATCCTGCGTTGATGCCTTTGTATGCATGTATTCAATCTA
AGCAGGCTTTCACTTTCTCGCCAACTTACAAGGCCTTTCTGTGTAAACAATACCTGAACCTTTACCCCGTTGCCCG
GCAACGGCCAGGTCTGTGCCAAGTGTTTGCTGACGCAACCCCCACTGGCTGGGGCTTGGTCATGGGCCATCAGC
GCATGCGTGGAACCTTTTCGGCTCCTCTGCCGATCCATACTGCGGAACTCCTAGCCGCTTGTTTTGCTCGCAGCAG
GTCTGGAGCAAACATTATCGGGACTGATAACTCTGTTGTCCTATCCCGCAAATATACATCGTTTCCATGGCTGCTAG
GCTGTGCTGCCAACTGGATCCTGCGCGGGACGTCCTTTGTTTACGTCCCGTCGGCGCTGAATCCTGCGGACGACC
CTTCTCGGGGTCGCTTGGGACTCTCTCGTCCCCTTCTCCGTCTGCCGTTCCGACCGACCACGGGGCGCACCTCTCT
TTACGCGGACTCCCCGTCTGTGCCTTCTCATCTGCCGGACCGTGTGCACTTCGCTTCACCTCTGCACGTCGCATGG

DR1

AGACCACCGTGAACGCCCACCAAATATTGCCCAAGGTCTTACATAAGAGGACTCTTGGACTCTCAGCAATGTCAAC

GACCGACCTTGAGGCATACTTCAAAGACTGTTTGTTTAAAGACTGGGAGGAGTTGGGGGAGGAGATTAGGTTAA

AGGTCTTTGTACTAGGAGGCTGTAGGCATAAATTGGTCTGCGCACCAGCACC //BCP

PreC mRNA transcription initiation site

ATGCAACTTTTTCACCTCTGCCTAA //Redundant sequence (nt 1816-1840; 25bp)

pgRNA transcription initiation site

CCCCAACTGGGGTAACCTTTGGGCTCCCCGGGCGCG //attR (36bp)

The attR recombination site (highlighted with red) is located downstream of the PreC/pgRNA transcription initiation site within the basic core promoter (BCP; highlighted with pink), by which do not disrupt pgRNA and 3.5kb mRNA (which encodes PreC/C and P genes) transcription. PreC mRNA and pgRNA transcription initiation sites are marked using blank box.

Fig S1. Prolonged HBV replication and expression in rcccDNA hydrodynamic-injected mice.

Method

Female immune-competent C57BL/6 and immune-deficient NOD/SCID mice (6 to 8 weeks of age) were purchased from Vital River Laboratories (Beijing, China). Both strains of mice were randomly divided into two groups (n = 5 each). The hydrodynamic procedure was used to inject approximate equimolars of rcccDNA (4 μg per C57BL/6 mouse; 5.7 μg per NOD/Scid mouse) and pTHBV2 plasmid (9 μg per C57BL/6 mouse; 13 μg per NOD/Scid mouse) into the mice within 5-8 seconds through the tail veins in a volume of phosphate-buffered saline (PBS) equivalent to 8% of the mouse body weight. Mice serum and livers were sampled at the indicated time point to evaluate HBV replication and expression.

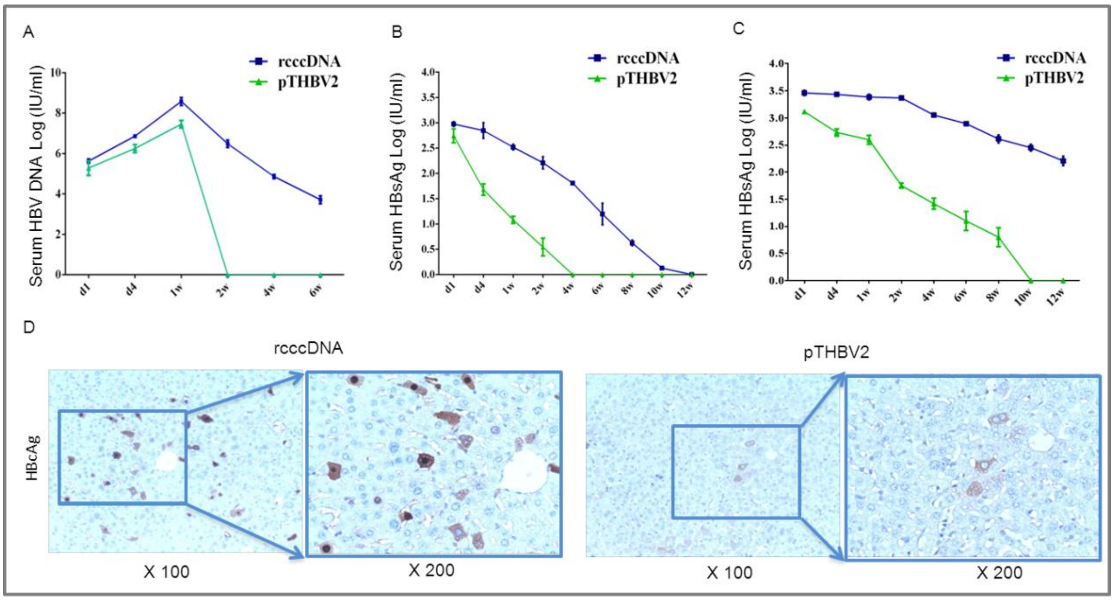

Fig S1. Prolonged HBV replication and expression in rcccDNA hydrodynamic-injected mice. Circulating viral DNA (A) or HBsAg (B) in the C57BL/6 mice treated with equimolar rcccDNA (4 μg per mouse, n = 5) and pTHBV2 (9 μg per mouse. n = 5). (C) Circulating HBsAg dynamics in NOD/SCID mice. The mice treated with equimolar amount of rcccDNA (5.7 μg per mouse) and pTHBV2 (13 μg per mouse), respectively. n = 5 for each group. (D) Immunohistological illustration of liver HBcAg at week 8 post-HBV vector injection in C57BL/6 mice.

In the immune-competent C57BL/6 mice injected with pTHBV2 plasmid, the circulating viral DNA was cleared within 2 weeks and HBsAg within 3 weeks. In contrast, the serum viral DNA persisted for 8 weeks (Fig S1A) and HBsAg for up to 10 weeks (Fig S1B) in the rcccDNA injected C57BL/6 mice. Using immunohistological staining, intensive HBcAg was illustrated in the mice liver 8 weeks after rcccDNA injection (Fig S1D left); in contrast, it was barely visible in the pTHBV2 group (Fig S1D right). We detected similar levels of circulating viral DNA in the first 7 days after injection of the two types of viral genomes, suggesting a similar viral replication activity in the early stage of viral genome transfection.

In the immune-deficient NOD/SCID mice injected with pTHBV2 plasmid (Fig S1C), the expression of circulating HBsAg was began declining at week 6 and became undetectable at week 10 post-injection. Whereas, a steadily high-level of HBsAg lasted for more than 3 months, in the rcccDNA injected NOD/SCID mice.
